# Complete resection for pleomorphic lung cancer with a high serum IL-6 level: a case report

**DOI:** 10.1186/s40792-016-0232-8

**Published:** 2016-09-23

**Authors:** Soichi Oka, Hiroki Matsumiya, Shuichi Shinohara, Taiji Kuwata, Masaru Takenaka, Yasuhiro Chikaishi, Ayako Hirai, Naoko Imanishi, Koji Kuroda, Hidetaka Uramoto, Fumihiro Tanaka

**Affiliations:** Second Department of Surgery, School of Medicine, University of Occupational and Environmental Health, 1-1 Iseigaoka, Yahatanishi-ku, Kitakyushu, 807-8555 Japan

## Abstract

**Background:**

Pleomorphic lung cancer cells have been reported to produce cytokines, resulting in systemic reactions. Recently, the autonomous production of hematopoietic cytokines (granulocyte colony-stimulating factor [G-CSF], granulocyte-macrophage colony-stimulating factor [GM-CSF], and interleukin-6 [IL-6]) was observed in some of these patients.

**Case presentation:**

We herein report a case of complete resection of right pleomorphic lung cancer producing IL-6. The patient had a high-grade fever before surgery, and a blood examination showed high IL-6 and CRP levels in the serum. After surgery, the patient no longer had a fever, and the elevated serum IL-6 levels had dropped to values less than those before the operation. Immunohistochemically, the carcinoma cells were faintly or focally positive for IL-6 and negative for G-CSF.

**Conclusions:**

The symptoms in the present case were dramatically improved by surgery. In addition, an immunohistochemical examination showed that the cancer cells were positive for IL-6.

## Background

Lung cancer cells have been reported to produce several cytokines and growth factors, especially interleukin (IL)-6 and granulocyte colony-stimulating factor (G-CSF), resulting in various systemic reactions [1–4]. We experienced a case of pleomorphic lung cancer producing IL-6.

## Case presentation

**Fig. 1:**
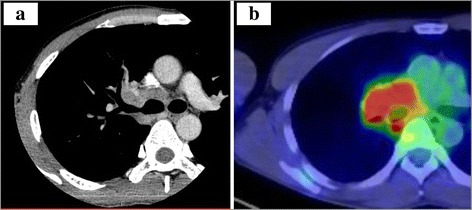
Preoperative imaging studies. Computed tomography of the chest showing the localization of the tumor. The tumor is located in the right hilar. The tumor surrounds the right upper bronchus (**a**). 18-fluorodeoxyglucose (FDG) positron emission tomography/computed tomography shows moderate FDG uptake within the tumor (**b**). The *pink-colored* area indicates that the maximum standardized uptake value is 8.1 or more

A 39-year-old Japanese male presented at our hospital due to an abnormal chest computed tomography (CT) scan showing a 35 × 25 × 25 mm tumor located in the right hilar region. This tumor was suspected to have invaded the right main bronchus and right main pulmonary artery (Fig. 1). 18-fluorodeoxyglucose (FDG) positron emission tomography/computed tomography showed moderate FDG uptake within the tumor (Fig. 1). There was no significant distant metastasis. The patient had general fatigue and appetite loss and had a fever for about 3 weeks. He had no remarkable medical history. His tumor marker levels were not elevated. The findings from laboratory tests, including a panel of antibodies for autoimmune processes, and an immunohistochemical analysis for lymphoma were all negative. His serum IL-6 levels were markedly high (28.2 pg/ml [<2.41]), but his serum G-CSF levels were not.

We performed endobronchial ultrasound-guided transbronchial needle aspiration (EBUS-TBNA). The tumor was found to be a carcinoma (suspected giant cell carcinoma; clinical-T2aN1M0 stage IIA), so we performed right upper sleeve lobectomy, because of the tumor invading the base of the right upper lobe bronchus.

The surgery was successful, with no major postoperative complications. The patient was discharged in a healthy condition 10 days after the surgery. He no longer had a fever, and his post-surgery 9-day serum IL-6 level was 10.9 pg/ml, which was lower than that before the operation.

**Fig. 2:**
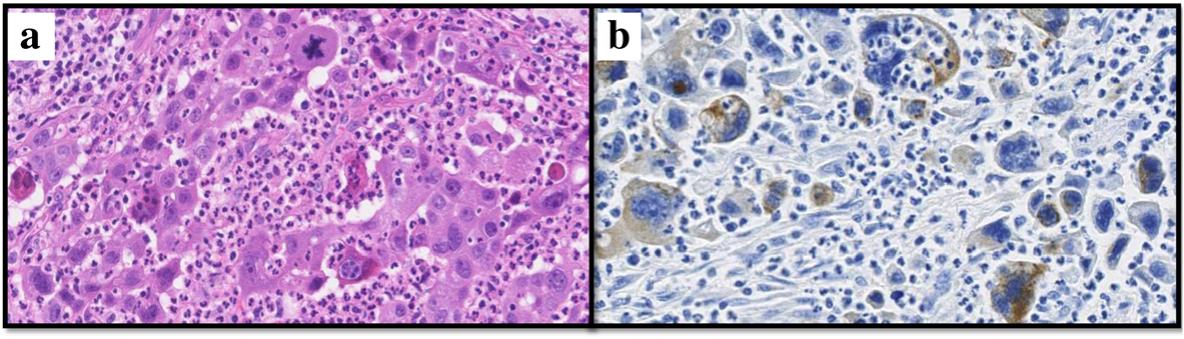
The figure shows the pathological findings. The tumor shows a proliferation of atypical epithelial cells having pleomorphic nuclei and prominent nucleoli arranged in a lepidic, acinar, or solid growth fashion (adenocarcinoma component), admixed with bi-nucleated or multinucleated cells with occasional emperipolesis (giant cell carcinoma component) with stromal, consistent with pleomorphic carcinoma (**a**). Immunohistochemically, the carcinoma cells are faintly or focally positive for IL-6 (**b**)

Pathologically, the tumor showed proliferation of atypical epithelial cells having pleomorphic nuclei and prominent nucleoli arranged in a lepidic, acinar, or solid growth fashion (adenocarcinoma component), mixed with bi- and multinucleated cells with occasional emperipolesis (giant cell carcinoma component) and stroma, findings which were consistent with a pleomorphic carcinoma. Immunohistochemically, the carcinoma cells were faintly or focally (50 % tumor cells positive) positive for IL-6 but negative for G-CSF (Fig. 2). Giant cell proportions were 30 % in this tumor. IL-6 positive carcinoma cells were 50 % in these tumor cells which include the adenocarcinoma component. There were no lung abscesses and inflammatory changes. Finally, this case’s pathological stage was T2aN1M0 stage IIA pleomorphic carcinoma. We performed adjuvant chemotherapy (three cycles of cisplatin and pemetrexed) after surgery. This patient is alive and recurrence-free 1 year after surgery.

## Conclusions

This report has two important implications. First, the symptoms in this case were dramatically improved by surgery. The patient initially presented with a high-grade fever and elevated C-reactive protein (CRP) levels and serum concentrations of IL-6. Following right upper sleeve lobectomy, however, his serum IL-6 level was 10.9 pg/ml, which was lower than pre-operation. CRP-elevated concentrations normalized, and the fever dissipated after surgery.

Second, the findings from an immunohistochemical examination showed that the cancer cells were positive for IL-6. The elevated IL-6 levels in this patient may have contributed to his high-grade fever and high CRP levels. This case was pleomorphic carcinoma. These cytokines have been reportedly produced by lung cancer, especially by large cell carcinomas. There were a few cases reported that pleomorphic lung cancer produced IL-6 [3]. Thus, our report is valuable.

Kasuga et al. reported that tumor-related leukocytosis is an ominous prognostic sign in patients with lung cancer [5]. Further, Mochizuki et al. reported that pleomorphic carcinoma should be considered as an aggressive disease and massive necrosis should be routinely reported and used as a factor in clinical assessments [6]. In our case, this patient has experienced no recurrence and no leukocytosis for 1 year after his surgery. And, pathologically, this tumor has a few necrosis areas. However, subsequent careful follow-up should be continued.

Finally, several reports described patients of the pleomorphic lung cancer to have a fever and cancer cell producing IL-6 cytokine [3, 5]. Therefore, measurement of IL-6 may be help to diagnose the tumor before therapy and predict the recurrence.

## Abbreviations

CRP: C-reactive protein
CT: Computed tomography
G-CSF: Granulocyte colony-stimulating factor
IL: Interleukin
